# Research Progress on the Antiosteoarthritic Mechanism of Action of Natural Products

**DOI:** 10.1155/2021/7714533

**Published:** 2021-09-30

**Authors:** Mingzhu Gao, Chun Chen, Qiaoyan Zhang, Jun Bian, Luping Qin, Leilei Bao

**Affiliations:** ^1^Department of Pharmacy, Eastern Hepatobiliary Surgery Hospital, Second Military Medical University, Shanghai 200438, China; ^2^Jiangxi University of Traditional Chinese Medicine, Nanchang 330000, China; ^3^College of Pharmacy, Zhejiang Chinese Medical University, Hangzhou 310000, China; ^4^Department of Pharmacy, Changhai Hospital, Second Military Medical University, Shanghai 200433, China

## Abstract

**Background:**

Osteoarthritis (OA) is a clinical joint degenerative disease, the pathogenic factors of which include age, obesity, and mechanical injury. Its main pathological features include cartilage loss, narrowing of joint space, and osteophyte formation. At present, there are a variety of treatment methods for OA. Natural products, which are gradually being applied in the treatment of OA, are advantageous as they present with low toxicity and low costs and act on multiple targets.

**Methods:**

The terms “natural products,” “osteoarthritis,” and “chondrocytes” were searched in PubMed to screen the related literature in the recent 10 years.

**Results:**

We comprehensively introduced 62 published papers on 48 natural products involving 6, 3, 5, 12, 4, and 5 kinds of terpenoids, polysaccharides, polyphenols, flavonoids, alkaloids, and saponins, respectively (and others).

**Conclusion:**

The mechanisms of their anti-OA action mainly involve reducing the production of inflammatory factors, reducing oxidative stress, regulating the metabolism of chondrocytes, promoting the proliferation of chondrocytes, or inhibiting chondrocyte apoptosis. This article summarizes the anti-OA activity of natural products in the last 10 years and provides candidate monomers for further study for use in OA treatment.

## 1. Introduction

Osteoarthritis (OA) is a common orthopedic disease worldwide. It is characterized by articular cartilage loss, subchondral bone changes, osteophyte formation, and synovitis [1]. Genetic factors, mechanical damage, age, and obesity are risk factors for OA. Articular cartilage injury includes degradation of the extracellular matrix (ECM) and apoptosis of chondrocytes. Abnormally elevated inflammatory factors in the synovial fluid interact with receptors on the membrane of chondrocytes and interfere with the normal functioning of some signaling pathways in these cells. They equally mediate the transcription of specific genes, causing apoptosis or degradation of the ECM of chondrocytes, leading to the further progression of OA [2].

The main goals of the treatment of OA under the modern medical system are to improve or maintain joint function, prevent the progression of OA, and reduce pain. This treatment includes drug and nondrug therapy. Drug therapy is mostly symptomatic and involves the use of oral nonsteroidal anti-inflammatory drugs (NSAIDs), such as acetaminophen, celecoxib, and diclofenac, topical capsaicin cream, intra-articular glucocorticoid injections, and opioid drugs such as codeine, for moderate to moderately severe pain. Intra-articular injection of steroids or hyaluronic acid and its derivatives is another treatment option. There also exist other treatment options such as joint replacement surgery and physical exercise.

Recently, there have been increasing reports of natural animal and plant components that possess anti-inflammatory, antiapoptotic, proliferation-promoting, oxidation-inhibiting, and other pharmacological effects in OA, especially on articular chondrocytes [3]. Also, natural products should be assessed by modern western medicine methods. In this paper, the main monomers occurring in natural products with reported therapeutic effects in OA in the recent years are summarized. This can provide candidate monomers for the drug therapy of OA.

## 2. Materials and Methods

The terms “natural products,” “osteoarthritis,” and “chondrocytes” were searched in PubMed to screen the related literature in the recent 10 years. Then, it is classified according to structure and pharmacological action.

## 3. Antiosteoarthritic Action of Natural Products

Some studies have found that natural products possess better anti-OA effects than traditional drugs, such as glucosamine and chondroitin. Researchers are increasingly interested in the use of natural products in the treatment of OA [4]. At present, terpenoids, polyphenols, flavonoids, quinones, phenolic acids, polysaccharides, alkaloids, saponins, and other natural products have been found to have antiosteoarthritic effects.

### 3.1. Terpenoids

**Figure 1:**
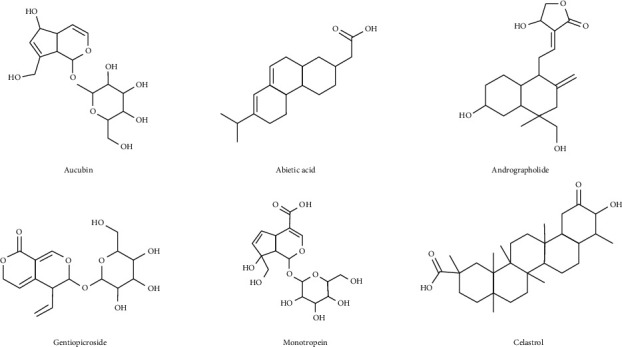
Structural formula of terpenoids with antiosteoarthritis effects from natural products.

Terpenoids are basic substances involved in plant growth and metabolism [5]. Terpenoids elicit anti-inflammatory and anticancer effects by acting on multiple targets. They can inhibit the activation of NOD-like receptor family pyrin domain containing 3 (NLRP3) inflammatory body, so as to regulate the pathogenicity of inflammatory diseases, such as colitis and gouty arthritis. The combined inhibition of TLR4-The noncanonical nuclear factor-kappa B (NF-*κ*B) and NLRP3 inflammatory activation process may make terpenoids a very promising candidate for the development of safe and effective new therapies for NLRP3 inflammatory diseases. D-limonene belongs to monoterpenoids. In the orthotopic mouse model of human gastric cancer, d-limonene may inhibit tumor growth and metastasis through its antiangiogenesis, antiapoptosis, and antioxidant effects. Further evidence from a phase I clinical trial showed that one breast cancer patient was partially relieved, and three CRC patients were stable for more than six months. Poria acid, a wool triterpene derived from Poria cocos, shows cytotoxicity to human lung cancer A549 cells, human prostate cancer DU145 cells, and colon cancer HT29 cells and induces apoptosis of DU145 and LNCaP prostate cancer cells [6, 7]. Many studies have found that terpenoids possess considerable antiosteoarthritic effects such as aucubin, abietic acid, andrographolide, gentiopicroside, monotropein, and celastrol. The specific mechanism of anti-OA is described in Section 3. The chemical structures shown in Figure 1 are terpenoids with antiosteoarthritic properties.

### 3.2. Polysaccharides

Polysaccharides are important active compounds in traditional Chinese medicine (TCM). Polysaccharides obtained from TCMs exhibit a variety of biological activities such as reducing the concentration of inflammatory factors and increasing resistance to viruses and tumors. The polysaccharide extracted from *Gelidium cartilagineum* has anti-influenza B and mumps virus activity. *Astragalus* polysaccharide has anti-infectious bursal disease virus, porcine circovirus type 2 virus, duck hepatitis A virus, human hepatitis B virus, porcine reproductive and respiratory syndrome virus, and classical swine fever virus. *Lycium barbarum* polysaccharides and *Angelica sinensis* polysaccharides kill tumor cells by inducing apoptosis. *Radix Hedysari* polysaccharides and *Achyranthes bidentata* polysaccharides can inhibit cell cycle and play an antitumor role. Traditional Chinese medicine polysaccharides do not act on tumor cells, but play a role by activating the immune system, such as *Sophora flavescens* polysaccharide and *Sanguisorba officinalis* polysaccharide [8]. Polysaccharides are generally safe and nonirritating and have been widely used in the treatment of OA [9]. In recent years, in-depth research has been carried out on the antiosteoarthritic activities of polysaccharides, such as angelica polysaccharide, *Achyranthes bidentata* polysaccharide, and others. The specific mechanism of anti-OA is described in Section 3.

### 3.3. Polyphenols

**Figure 2:**
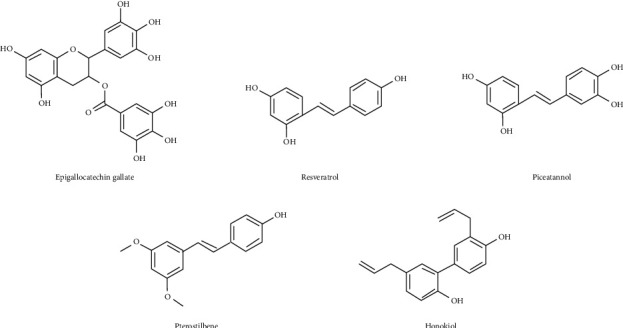
Structural formula of polyphenols with antiosteoarthritis effects from natural products.

Polyphenols are natural antioxidants that can reduce oxidative damage to lipids, proteins, enzymes, carbohydrates, and DNA in living cells and tissues. Resveratrol was found to stimulate endothelial production of nitric oxide, reduce oxidative stress, inhibit vascular inflammation, and prevent platelet aggregation [10]. Recent studies have found that some natural polyphenols have antiosteoarthritic effects such as epigallocatechin gallate, resveratrol, piceatannol, pterostilbene, and honokiol [11–15]. Their structures are shown in Figure 2. The specific mechanism of anti-OA is described in Section 3.

### 3.4. Flavonoids

**Figure 3:**
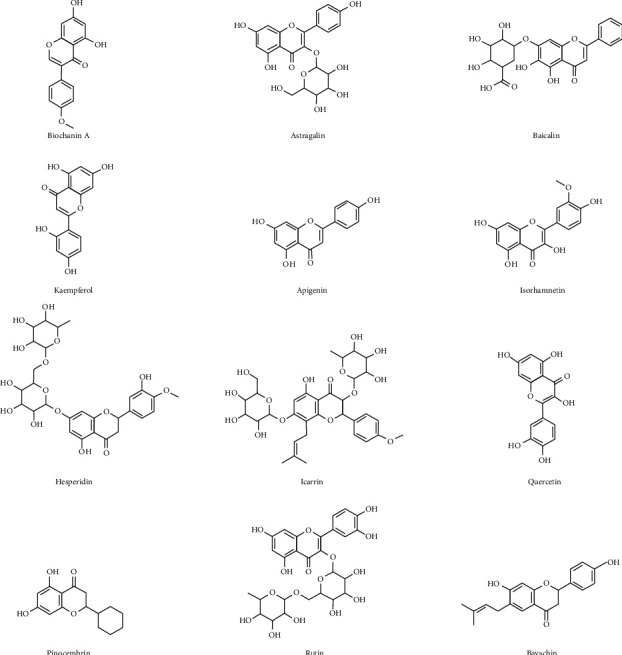
Structural formula of flavonoids with antiosteoarthritis effects from natural products.

Flavonoids are secondary metabolites that have an important anti-inflammatory role via reducing the activation of inflammatory bodies. The effects of quercetin on the inflammasome during fructose-induced hyperuricemia and dyslipidemia in rats were studied. It was found that quercetin improved renal inflammation and disease symptoms by reducing the renal expression of NLRP3 inflammasome components (such as NLRP3, ASC, and caspase-1) and reducing the levels of IL-1, IL-18, IL-6, and TNF- *α* in kidney and serum of fructose fed rats [16]. Flavonoids are widely found in fruits, vegetables, nuts, cereals, and so on [17]. Studies have found that flavonoids, such as biochanin A, astragalin, baicalin, kaempferol, apigenin, isorhamnetin, hesperidin, icariin, quercetin, pinocembrin, rutin, and bavachin, the chemical structures of which are shown in Figure 3, have therapeutic effects on OA. Their structures are shown in Figure 2. The specific mechanism of anti-OA is described in Section 3.

### 3.5. Alkaloids

**Figure 4:**
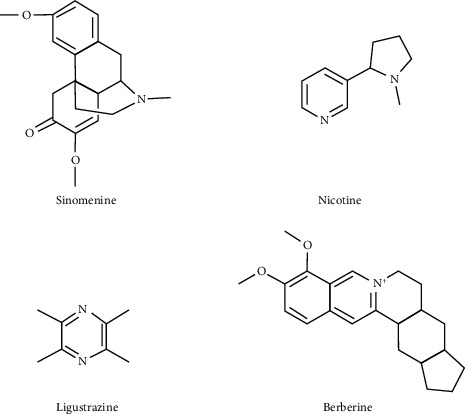
Structural formula of alkaloids with antiosteoarthritis effects from natural products.

Alkaloids, a type of natural components used widely in traditional Chinese medicine, regulate cell morphology, apoptosis, and autophagy to prevent and treat cancer. For example, matrine-induced stress destroyed the proliferation and migration of HepG2 cells. In addition, matrine promotes apoptosis of hepatoma cells, which is related to extracellular signal regulated kinase (ERK). In addition, lycorine treatment can also significantly reduce cell viability by inducing HCC cell cycle arrest in G2/*M* phase and reducing the expression of cyclin A, B1, C2, and dependent kinase [18]. Sinomenine, nicotine, tetramethylpyrazine, and berberine, the structures of which are shown in Figure 4, are alkaloids that can relieve OA. The specific mechanism of anti-OA is described in Section 3.

### 3.6. Saponins

Saponins are natural glycosides that possess neuroprotective effect. Ginsenoside Rd has a protective effect on focal cerebral ischemia in aged mice, which may be related to the weakening of redox imbalance. Ginsenoside Rd has the characteristics of penetrating the complete blood-brain barrier and has a protective effect on transient and permanent middle cerebral artery occlusion in the rat experimental stroke model. Ginsenoside Rd has been shown to partially improve neurological deficits in patients with acute ischemic stroke in a multicenter, randomized, double-blind, placebo-controlled phase II trial [19, 20]. Saponins found in some traditional Chinese medicinal plants, such as *Achyranthes bidentata, Panax ginseng, Gynostemma pentaphyllum, Polygala tenuifolia,* and *Hedera helix,* have antiosteoarthritic effects [21–25]. The specific mechanism of anti-OA is described in Section 3.

### 3.7. Others

**Figure 5:**
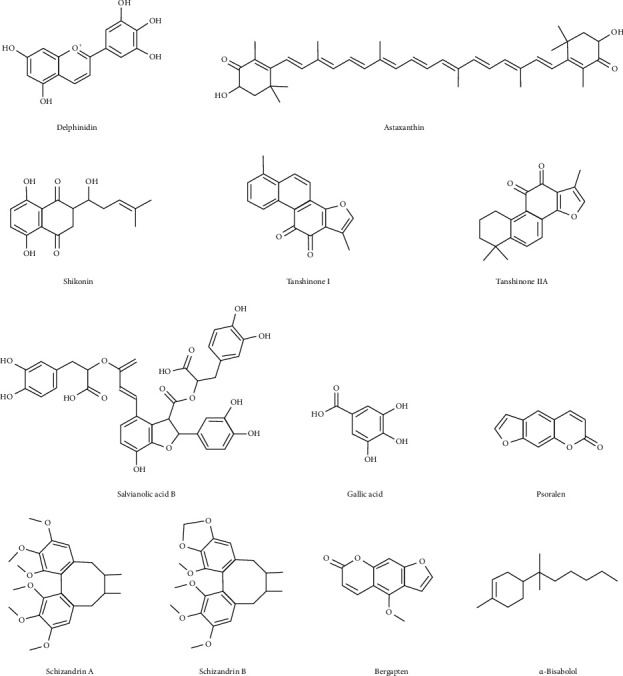
Structural formula of others with antiosteoarthritis effects from natural products.

Natural pigments, such as delphinidin, astaxanthin, and shikonin, have also been found to relieve OA in recent years. In addition, quinones and phenolic acids found in *Salvia miltiorrhiza* were found to be beneficial in treating OA [26]. Schisandrin is a lignan that can also relieve OA [27]. Coumarins, such as psoralen and bergapten, also have antiosteoarthritic activities [28, 29]. Polyoxypregnane glucosides and *α*-bisabolol are also natural products with comparable efficacy in OA [30, 31]. Their structures are shown in Figure 5.

## 4. Mechanisms of Action of Natural Products in OA Treatment

OA is accompanied by inflammatory response and inflammation is usually associated with mitochondrial damage. Production of reactive oxygen species (ROS) after mitochondrial injury promotes mitochondrial autophagy and leads to abnormal cell function. After chondrocyte injury, the increased secretion of matrix metalloproteinases (MMPs) and disintegrin and metalloproteinase with thrombospondin motifs (ADAMTS) leads to ECM degradation, promotes chondrocyte apoptosis, and accelerates the process of OA. The decrease of chondrocytes leads to further degradation of articular cartilage and the production of MMPs, synovial angiogenesis, and inflammatory factors and promotes further destruction of cartilage.

The effects of natural products on OA are mainly concentrated in chondrocytes. They can improve cytokine content and signaling pathways in chondrocytes, resulting in the reduction in the production of inflammatory factors, reduction in oxidative stress, regulation of chondrocyte metabolism, promotion of chondrocyte proliferation, or inhibition of chondrocyte apoptosis. In the following sections, the therapeutic effects of natural products on OA are presented in detail.

### 4.1. Reduction of the Production of Inflammatory Factors

Inflammatory factors promote OA by affecting the normal physiological functions of chondrocytes. The accumulation of inflammatory factors such as IL-1*β*, TNF-*α*, NO, PGE2, and COX-2 can induce the production of more NO by chondrocytes and damage the mitochondrial respiratory chain, leading to apoptosis and degradation of the ECM of chondrocytes. Reducing the production of these inflammatory components is conducive for normal chondrocyte growth [2].

#### 4.1.1. Terpenoids

Aucubin is an iridoid compound found in *Eucommia ulmoides, Plantago asiatica,* and *Rehmannia glutinosa.* It is the main active component of these plants and has anti-inflammatory, antioxidant, and liver protection functions. In H_2_O_2_-treated chondrocytes, aucubin reduced cell damage [32]. Abietic acid, the main component of *Colophonium*, can elicit an anti-inflammatory action by inhibiting the nuclear transcription factor-kappa B (NF-*κ*B) and mitogen-activated protein kinase (MAPK) signaling pathways [33].

#### 4.1.2. Polysaccharides and Polyphenols

*Angelica sinensis* polysaccharides can prevent chondrocyte apoptosis and suppress the gene expression of inflammatory factors such as IL-1*β* and TNF-*α* [34]. Leong et al. found that *Epigallocatechin gallate* combined with avocado soybean unsaponifiables could mitigate OA [35]. In addition, honokiol can suppress the IL-1b-triggered activation of the IkappaB kinase (IKK)/I-kappa-B-alpha (IkB*α*)/NF-kB signaling pathway and elicit anti-inflammatory effects [36].

#### 4.1.3. Flavonoids

Biochanin A, which is similar to estradiol, can bind to estrogen receptors and reduce cartilage ECM degradation through its anti-inflammatory effects [37]. Baicalin and astragaloside are natural saponins. Baicalin reduces inflammatory injury through the downregulation of miR-126, thereby blocking the activation of the NF-*κ*B signaling pathway [38].

#### 4.1.4. Alkaloids and Saponins

Sinomenine, isolated from *Sinomenium acutum*, is commonly used in the treatment of rheumatism. Wu et al. [39, 40] found that sinomenine, as well as resveratrol, can reduce the production of inflammatory factors via nuclear factor E2-related factor 2(Nrf2)/heme oxygenase 1(HO-1) and NF-*κ*B signaling. Nicotine is an effective acetylcholine receptor agonist, but has no clinical application value at present. The expression of IL-6 and IL-1*β* was found to be inhibited by nicotine in the chondrocytes of OA patients [41]. Astragaloside treats OA through its anti-inflammatory and antiapoptosis effects [42].

#### 4.1.5. Others

*Salvia miltiorrhiza* contains water-soluble and lipid-soluble components. The lipid-soluble components tanshinone IIA and salvianolic acid B have similar effects in reducing inflammatory factors [43, 44]. Astaxanthin is a carotenoid with strong antioxidant effects. When combined with hyaluronic acid and krill oil, it can reduce inflammatory reaction [45]. Delphinidin, one of the six main monomer forms of anthocyanins, has a suppressive action on the expression of COX-2 by inducing the production IL-1*β* and PGE2 and activating the NF-*κ*B signaling pathway [46].

Bergapten, widely found in fruits, limits the progression of OA by regulating the acidic leucine‐rich nuclear phosphoprotein‐32A (ANP32A)/ataxia‐telangiectasia mutated (ATM) signaling pathway [47]. *α*-Bisabolol has a variety of biological activities, one of which is anti-inflammation, elicited by blockage of the NF-*κ*B, p38, and c-Jun N-terminal kinase (JNK) signaling pathways [48].

### 4.2. Reduction of Oxidative Stress

Excessive oxygen free radicals in the joint accelerate the oxidative stress reaction, resulting in serious damage to chondrocytes and the ECM, thus favoring the advancement of OA. Malondialdehyde (MDA) and superoxide dismutase (SOD) are two important indicators for the measurement of the oxidative stress response. They participate in the intracellular signal transduction mechanism. Once there is an imbalance in their concentrations, the oxidative stress response is aggravated.

#### 4.2.1. Terpenoids and Polysaccharides

Andrographolide has antioxidant and free radical scavenging effects related to the Keap1-Nrf2-antioxidant response element (ARE) signal pathway [49]. In addition, Ajeeshkumar et al. [50] found that the proteoglycan extracted from thorn shark cartilage could maintain the activity of SOD in an OA rat model.

#### 4.2.2. Polyphenols

Wen et al. [51] found that gallic acid can reduce ROS content and increase SOD content in rabbit chondrocytes exposed to AGEs. In addition, pterostilbene attenuates ROS production in the mitochondria and cytoplasm of IL-1*β*-induced chondrocytes [52].

#### 4.2.3. Flavonoids

Pan et al. [53] found that baicalin can reduce oxidative stress and apoptosis in endplate-H_2_O_2-_induced chondrocytes. In another study, kaempferol, apigenin, and synovial membrane derived stem cells (SMMSCs) injected into the joints of OA rats increased SOD levels in the cartilage homogenate and decreased MDA levels [54]. In addition, Zhou et al. [55] found that isorhamnetin can reduce ROS production induced by RANKL and protect chondrocytes from oxidative stress, which leads to apoptosis. Hesperidin, a dihydroflavone glycoside found in the pericarp of *Citrus limon* and mandarin orange, inhibits oxidative stress in H2O2-induced rat chondrocytes [56].

#### 4.2.4. Alkaloids, Saponins, and Others

Ligustrazine is the main active component in *Ligusticum chuanxiong* rhizomes. In IL-1*β*-induced chondrocytes, ligustrazine has anti-inflammatory and antioxidant effects, regulating SOX9 concentration and the NF-*κ*B signaling pathway [57]. Advanced glycation end products (AGEs) can induce physiological and pathological reactions in cells. Xu et al. [58] found that Hederacoside-C can downregulate oxidative stress in OA. Lee et al. [59] observed that delphinidin could activate the Nrf2 signaling pathway, promote autophagy of chondrocytes, and protect them against oxidative stress.

### 4.3. Regulation of Chondrocyte Metabolism

Abnormal metabolism in chondrocytes, which is characterized by damage to the cartilage ECM, including the degradation of proteoglycan and collagen, can also promote or aggravate OA. Overexpression of MMPs and ADAMTS can lead to the degradation of proteoglycan and collagen. However, the tissue inhibitor of metalloproteinase (TIMP) can inhibit the activity and reduce the degradation of proteoglycan.

#### 4.3.1. Polyphenols and Alkaloids

Tang et al. [60] found that piceatannol, found in a number of food items, inhibited IL-1*β*-induced NF-*κ*B activation by activating the Nrf2/HO-1 pathway. Teng et al. [61] found that nicotine can activate the phosphatidylinositide 3-kinase (PI3K)/Akt and inhibit the NF-*κ*B signaling pathways in OA animal models. Berberine can promote cell survival and matrix production through the activation of the Akt/p70S6K/S6 signaling pathway [62].

#### 4.3.2. Flavonoids

Quercetin is widely distributed in the plant kingdom and has anti-inflammatory, antiviral, and antitumor pharmacological activities. It can maintain the integrity of the ECM of articular cartilage [63–65]. Chen et al. [66] showed that baicalin can reduce cartilage destruction. The TCM “*Morinda officinalis*” is the dried root of *Morinda officinalis*. Its iridoid component, Monotropein, can significantly reduce the gene expression of MMP-3 and MMP-13 in IL-1*β*-induced chondrocytes [67]. Zhang et al. [68] found that pinocembrin also acts on the ECM by blocking NF-kB signaling pathway. Wu et al. [69] found that at the mRNA and protein levels, Biochanin A can downregulate the expression of MMPs, upregulate the expression of TIMP-1, and block the NK-*κ*B signaling pathway. Bavachin, a phytoestrogen, can promote the effective synthesis and antidecomposition of ECM [70].

#### 4.3.3. Saponins

Icariin, an extract from dried stems and leaves of *Epimedium*, can inhibit the p38, JNK, and *β*-catenin signaling pathways in IL-1*β*-induced chondrosarcoma SW1353 cells and OA rat models [71]. Gentiopicroside is the main active component of *Gentianaceae* plants. It can enhance MAPK signaling pathways that prevent P38, extracellular regulated kinases (ERK), and JNK phosphorylation [72]. *Panax ginseng* contains a variety of saponins. Ginsenoside Rg1 can reduce the degradation of proteoglycan and type II collagen [73]. Other studies have found that gypenoside reduces the activation of signaling pathways, thus protecting cartilage [74]. Tenuigenin, found in *Polygala tenuifolia,* also suppresses the expression of IL-1*β*-induced MMP-1, MMP-3, and MMP-13 [75].

#### 4.3.4. Others

*Schisandra chinensis* contains many kinds of lignans, including schisandrin A and schisandrin B. In OA treatment, schisandrin B and schisandrin A have similar pharmacological effects. They can inhibit the production of MMPs and ADAMTS-5 in IL-1*β*-induced chondrocytes and upregulate the gene expression of type II collagen and proteoglycan [76]. Furthermore, Astaxanthin, which targets the chondrocyte matrix, is a potential therapeutic drug for OA [77]. Furthermore, Tanshinone I can reduce the degradation of type II collagen and proteoglycan, upregulate the gene expression of (SRY)-related high-mobility group (HMG) box 11 (SOX11), downregulate the gene expression of MMP-13, and suppress the p-NF-*κ*B signaling pathway, reducing cartilage damage [78].

Shikonin is the main active component of *Lithospermum erythrorhizon.* It can adjust the equilibrium between the synthesis and degradation of cartilage [79]. Moreover, the polyoxypregnane glycoside from *Dregea volubilis* extracts may inhibit cartilage degradation in OA and may be used as a nutritive substance for maintaining joint integrity and function [80]. Psoralen, which has estrogen-like effects, protects chondrocytes and reduces the gene expression of MMPs and ILs [81].

### 4.4. Promotion of Chondrocyte Proliferation or Inhibition of Apoptosis

One of the characteristics of OA is chondrocyte apoptosis. Many genes and signaling pathways in the joint can affect the proliferation and apoptosis of chondrocytes. Once these genes and signaling pathways are abnormally expressed, they inhibit the proliferation of chondrocytes and promote their apoptosis. While autophagy can help in maintaining the normal physiological state of chondrocytes, its reduction can also promote injury to chondrocytes. Bone marrow mesenchymal stem cells have strong differentiation abilities and can be induced to differentiate into chondrocytes in specific environments conducive for cartilage reconstruction [82].

#### 4.4.1. Terpenoids

In IL-1*β*-induced chondrocytes, aucubin elicits its action by inhibiting chondrocyte apoptosis [83]. Celastrol, which can be extracted from *Celastrus orbiculatus, Tripterygium wilfordii,* and other plants, can block the NF-*κ*B signaling pathway in vivo and in vitro, strengthening chondrocyte autophagy and increasing resistance to apoptosis. Resveratrol also has the same effect [84, 85].

#### 4.4.2. Polysaccharides

Yu et al. have found that polysaccharides in *Radix Achyranthis Bidentatae* can significantly upregulate the gene expression of Wnt-4, frizzled-2, and *β*-catenin, downregulate the gene expression of GSK-3*β*, promote the nuclear translocation of *β*-catenin, and activate the Wnt/*β*-catenin signaling pathway [86, 87]. Polysaccharides from *Cibotium barometz* can promote the transition of chondrocytes from the G1 to the S phase [88].

#### 4.4.3. Flavonoids

Rutin, a quercetin glycoside derivative, can block these signaling pathways in H_2_O_2_-treated chondrocytes [89]. In addition, astragalin elicits antiapoptotic effects by mediating the autophagy of chondrocytes [90].

#### 4.4.4. Alkaloids

Furthermore, berberine, found in *Rhizoma Coptidis,* can promote the proliferation of chondrocytes in vitro and activate the Wnt/*β*-catenin signaling pathway [91]. Zheng et al. [92] found that nicotine reduces chondrocyte apoptosis by activating the PI3K/Akt signaling pathway in IL-1*β*-induced chondrocytes.

#### 4.4.5. Saponins and Others

Triterpenoid saponins isolated from *Achyranthes bidentate* have significant effects on IL-1b-induced apoptosis [93]. Activation of p-ERK, MAPK, and p-JNK is associated with chondrocyte apoptosis. Additionally, Zheng et al. [94] found that the Wnt/*β*-catenin signaling pathway, which can be activated by psoralen, can promote chondrocyte proliferation.

## 5. Discussion

At present, the complexity of OA, which has no cure, is still not fully understood. In recent years, integrated genomics, proteomics, metabolics, and bioelectronics were used to explore OA chondrocytes [95]. Losing weight and practicing sporting activities can improve joint pain and function in OA patients. Oral and topical NSAIDs are the most common treatment for OA. Intra-articular injection of corticosteroids and hyaluronic acid is also used [96]. The etiology of OA is very complex, involving many targets, and the current drug treatment, though accompanied by many side effects, can only relieve pain. Natural products have lesser side effects and act on multiple targets, and, thus, have increasingly attracted the attention of researchers. The treatment of OA is highly beneficial, as it improves the quality of life of the patient and reduces the national economic burden.

Western medicine is mainly employed in the clinical treatment of OA [97]. Natural products have a long history of consumption in China, and there are few toxic reactions, but some also have serious toxic effects. However, the research on the efficacy of traditional Chinese medicine is slow, and now it needs more in-depth research. Natural products are worth exploring for future use. For this, we need to understand the interaction between natural products and the relationship between corresponding targets and signaling pathways. Many natural products have shown potential for use in treating OA in vivo and in vitro, but there is a lack of preclinical and clinical trial data. More work is needed to gradually move candidate natural products to clinical trials, so that more patients can benefit directly [5].

**Figure 6:**
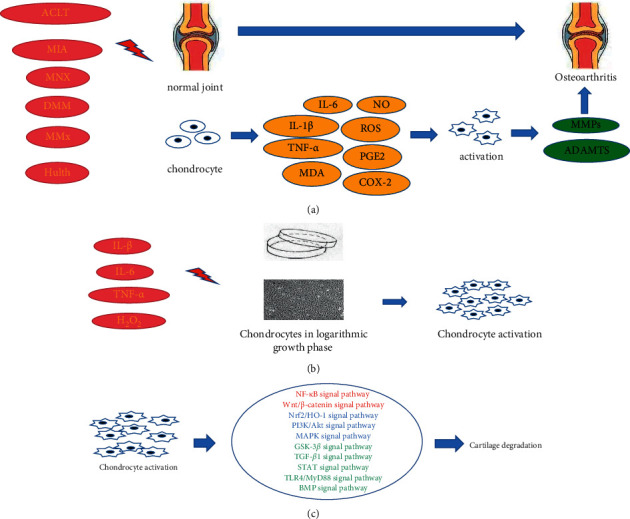
Animal models, cell models, and signaling pathways involved in the study of natural products with antiosteoarthritis effect. (a) Animal models used in the study of osteoarthritis. (b) Cell models used in the study of osteoarthritis. (c) Signaling pathways involved in the study of the mechanisms of natural products with antiosteoarthritis. The antiosteoarthritis mechanism of most natural products is related to the 2 signaling pathways marked in red. Some are related to the 3 signaling pathways marked in blue. A few are related to the 5 signaling pathways marked in green. ACLT: anterior cruciate ligament; MIA; injection of sodium iodoacetate; MNX; medial meniscectomy; DMM; destabilization of medial meniscus; MMxs; resection of medial menisci.

In this review, we summarized the literature on the antiosteoarthritic effects of natural products in the last decade. We comprehensively introduced 62 published papers on 48 natural products involving 6, 3, 5, 12, 4, and 5 kinds of terpenoids, polysaccharides, polyphenols, flavonoids, alkaloids, and saponins, respectively (and others). Research mainly focused on flavonoids, terpenoids, polyphenol, polysaccharides, alkaloids, and saponins. We summarize these natural products according to the structural classification, which are shown in Tables 1–7. We found that some natural products, such as baicalin, apigenin, icariin, quercetin, nicotine, and berberine, are studied more often than others. However, there are few available data for some natural products, such as polyoxypregnane glucoside, *α*-bisabolol, and saponins. Animal and cell models of OA are shown in Figure 6. Signaling pathways are involved in the antiosteoarthritic action of natural products (Figure 6). Most natural products are associated with the NF-*κ*B and Wnt/*β-*catenin signaling pathways. This article summarizes four aspects (inflammatory factors, oxidative stress, cell metabolism, and cell survival) to explain the antiosteoarthritic effects of natural products. A natural product may have multiple mechanisms in its antiosteoarthritic action. Different mechanisms are also interrelated; an increase in inflammatory factors will lead to the synthesis of MMPs, which will lead to cartilage tissue degradation. This study involved a variety of factors and signaling pathways, mainly TNF-*α*, IL-1*β*, MDA, SOD, ROS, NF-*κ*B, Nrf2/HO-1, PI3K/Akt, and Wnt/*β*-catenin. In summary, the pathogenesis of OA is complex, involving multiple factors, pathways, and mechanisms of pathological changes, which directly or indirectly lead to ECM degradation, cartilage damage, and subchondral bone hyperplasia. These factors and signaling pathways do not exist in isolation, but have interlocked and interlaced relationships. In conclusion, the potential therapeutic effects of natural products in OA are the result of multichannel and multitarget mechanisms, which need to be further investigated.

Due to the complex pathological nature of OA, its drug treatment is only symptomatic. We should look for effective anti-OA products from the natural products, using modern pharmacological methods to understand their mechanism of action, then screen for effective monomer components, and accumulate effective candidate drugs for the treatment of OA, which has important value for the development of the drug treatment for OA. Furthermore, researchers will continue to explore the mechanism of OA in the future, Whether the two studies can reach a synchronous level is a realistic problem we are facing. Therefore, the combination of research on the mechanism of OA and research on natural products is an important direction for future research. The association of natural products may produce synergism, reduce activity, and restrict detoxification. This provides a new direction for the future research of natural products.

A single ingredient is used in western medicine, and the natural product is also a single ingredient. They may have toxic and side effects. Acetaminophen and nonsteroidal anti-inflammatory drugs have potential adverse reactions in gastrointestinal tract, liver, heart, and kidney. These adverse reactions increase with the increase of dose and treatment duration [98]. In this double-blind randomized controlled trial, subjects with knee osteoarthritis received intra-articular triamcinolone acetonide or normal saline placebo every three months for two years. These results suggest that intra-articular triamcinolone acetonide cannot delay cartilage destruction, but may accelerate cartilage destruction, and the anti-inflammatory effect of steroids cannot play a role as a disease regulator [99]. Some natural products may also cause some side effects, such as hepatotoxicity, gastrointestinal discomfort, dizziness, fatigue, skin reaction, elevated serum aminotransferase level, male reproductive toxicity, and menstrual changes. Aconitine can induce a variety of arrhythmias and lead to death. Improper use of *Tripterygium wilfordii* may cause strong irritation of gastrointestinal tract, congestion, edema, and necrosis of gastrointestinal mucosa. It can also cause bleeding and necrosis of liver, kidney, heart, and other organs, which can directly damage the myocardium [100]. These studies show that when studying drugs for the treatment of osteoarthritis, both western medicine and natural products need to determine the dosage and toxic and side effects. Natural products need a long time in the process of treating diseases, so there are high requirements for their safety. As a way to explore new drugs, natural products should not only pay attention to the curative effect, but also pay attention to its toxicity.

Natural products are a valuable treasure house for drug selection, and their anti-OA effect is verified by experiments. This paper summarizes the anti-OA effect of natural products in the recent ten years, so as to prepare for the later research and development of lead compounds of anti-OA drugs.

## 6. Limitation

Most natural products in this review have not undergone clinical trials, and their safety cannot be guaranteed. More clinical data are needed to prove their safety. There is still a long way to go to be on the market. The description of natural products does not start from the direct cause of OA. At present, the research depth and breadth of natural products are still very limited, and the key research needs to be further explored.

## Figures and Tables

**Table 1:** Natural products of terpenoids with antiosteoarthritis function.

Compound	Source	Cell/animal model	Effect	Ref
Aucubin	Eucommia ulmoides, Plantago asiatica, Rehmannia glutinosa	IL-1*β*-induced porcine chondrocytes/chondrocytes of male C57BL/6 mice induced by DMM	Maintain ACAN and COL2A1 gene expressions, prevent IL-6 and MMP-13 gene upregulation, reduce the caspase-3 activity, and downregulate Bax, caspase-9 and caspase-3	[32]
Abietic acid	Colophonium	Human OA chondrocytes	Inhibited IL-1*β*-induced TNF-*α*, NO, PGE2 production, and COX-2 expression, suppressed MMP-1, MMP-3, and MMP-13 production	[33]
Andrographolide	Andrographis paniculata	Chondrocytes of SD rats induced by H_2_O_2_	Upregulate expression of SOD and decrease MDA activity, MMP-13, TIMP-1, and Il-6	[49]
Celastrol	*Celastrus orbiculatus*, *Tripterygium wilfordii*	Chondrocytes of SD rats/male SD rats induced by ACLT	Ameliorate IL-1*β*-induced chondrocyte apoptosis and increased the expression of LC3-II and Beclin-1 and, moreover, decrease the phosphorylation degree of I*κ*B*α* and P65	[84]

DMM: destabilization of medial meniscus; ACLT: anterior cruciate ligament.

**Table 2:** Natural products of polysaccharides with antiosteoarthritis function.

Compound	Source	Cell/animal model	Effect	Ref
Angelica sinensis polysaccharide	Angelica sinensis	Chondrocytes of male SD rats	Inhibit both apoptosis and expression of inflammatory cytokines (IL-1*β* and TNF-*α*), increase expression of anabolic gene (Col2a1, aggrecan, and SOX9), and decrease expression of catabolic gene (MMP-1, -3, and -9)	[34]
Cartilage proteoglycan of the thorn shark	Cartilage of Centrophorus	Female Wistar rats induced by MIA	Reduce the expressions of the inflammatory modulators including TNF-*α*, IL-1*β*, MMP-13, NOS2, IL-10, and COX-2	[50]
*Achyranthes* bidentata polysaccharide	Radix Cyathulae Bidentatae	Chondrocytes of male SD rats	Upregulate gene expression of cyclin D1, CDK4, and CDK6 and suppress NFATc1 transcriptional activity and the phosphorylation of MAPK pathways	[86,87]
Cibotium barometz polysaccharides	Cibotium barometz	Chondrocytes of male SD rats	Boost the mRNA and protein expression of cyclin D1, CDK4, and pRB	[88]

MIA: injection of sodium iodoacetate.

**Table 3:** Natural products of polyphenols with antiosteoarthritis function.

Compound	Source	Cell/animal model	Effect	Ref
Epigallocatechin gallate	Camellia sinensis	Horse chondrocytes induced by TNF-*α* and IL-1*β*	Reduce the content of COX-2, PGE2	[35]
Honokiol	Magnolia officinalis	Human OA chondrocytes induced by IL-1*β*	Inhibit expression/production of IL-6, COX-2/PGE2, and iNOS/NO	[36]
Resveratrol	Vitis vinifera, Arachis hypogaea, and Fructus Mori	Male C57BL/6 mice/chondrocytes of mice	Inhibit IL-1*β*, IL-6 ,and IL-18 expression levels, decrease caspase-3/9 activity, and stimulate HO-1/Nrf-2 signaling	[40,85]
Gallic acid	Cornus officinalis, Rheum palmatum, Punica granatum	Chondrocytes of male New Zealand rabbits	Decrease the content of ROS and increase the content of SOD	[51]
Pterostilbene	Semen Trigonellae, Vitis vinifera	Chondrocytes of SD rats	Inhibit the level of COX-2, iNOS, PGE2, and NO, prevent cartilage degeneration, and promot the nuclear translocation of Nrf2 in cartilage	[52]
Piceatannol	Seeds of Euphorbia lagascae	Human OA chondrocytes induced by IL-1*β*/C57BL/6 male wild-type (WT) mice induced by DMM	Attenuated cartilage degeneration and reduced type II collagen loss and MMP-13 levels	[61]

DMM: destabilization of medial meniscus.

**Table 4:** Natural products of flavonoids with antiosteoarthritis function.

Compound	Source	Cell/animal model	Effect	Ref
Biochanin A	*Trifolium* pratense	Chondrocytes of male SD rats/chondrocytes of Zealand rabbits induced by IL-1*β*/Zealand rabbits induced by ACLT	Inhibit expression of iNOS2, PGE2, and COX- 2, downregulate the expression of MMPs, and upregulate the expression of TIMP-1	[37,70]
Baicalin	*Astragalus* membranaceus	Human OA chondrocytes/male C57BL/6 mice OA surgery/male SD rats induced by H2O2	Reduce the production of IL-6, IL-8, TNF-*α*, NO, and PGE2, downregulate gene expression of MDA and upregulate gene expression of SOD, and downregulate the expression of MMP-3, MMP-13, and ADAMTS-5	[38,53,66]
Kaempferol, apigenin	Kaempferia galanga, Apium graveolens	Male SD rats induced by ACLT	Decrease the levels of MMP-13, MMP-3, TNF-*α*, IL-1*β*, iNOS, and MDA and increase the gene expression levels of SOD, collagen IIa1, aggrecan, and SOX9 genes	[54]
Isorhamnetin	Hippophae rhamnoides Linn	Chondrocytes of male C57BL/6J mice/male C57BL/6J mice induced by ACLT	Inhibited MAPK, NF‐*κ*B, and AKT signaling pathways	[55]
Hesperidin	Citrus limon and Mandarin orange	Chondrocytes of male SD rats/male SD rats with medial collateral ligament and medial meniscus removed	Decrease the formation of MDA and intracellular ROS, inhibit the Col2a1, aggrecan, and SOX9 gene expression, and increase the gene expression of caspase-3, IL-1*β*, TNF*α*, iNOS, and MMP-13.	[56]
Quercetin	Bupleurum, folium Mori, Sophora japonica, Crataegus pinnatifida	Chondrocytes of male SD rat induced by MNX/New Zealand rabbits induced by Hulth/male SD rats	Attenuate ROS generation and augment the glutathione (GSH) and glutathione peroxidase (GPx) expression levels, enhance mitochondrial membrane potential, oxygen consumption, (ATP) levels in mitochondria, but also increase the mitochondrial copy number. Suppress accumulation of NO, MMP-3, and MMP-13	[63–65]
Monotropein	Pyrola incarnata, *Morinda* officinalis	Kunming rats induced by Hulth	Decrease expressions of MMP-3 and MMP-13 and increase the expression of COL2A1	[67]
Bavachin	*Psoralea* corylifolia	Chondrocytes of SD rats	Upregulate gene induction of both aggrecan and collagen type II, major extracellular matrix components, inhibit the expression of iNOS, and downregulate the expression of NO, COX-2, and PGE2	[70]
Rutin	Flos Sophorae	Chondrocytes of male SD rats induced by H2O2	Activate SIRT1 and block NF-*κ*B/MAPK signaling pathway	[89]
Astragaloside IV	Scutellaria baicalensis	Human OA chondrocytes	Increase the protein expression of LC3-II/I and decrease P62/SQSTM1 expression	[90]

ACLT: anterior cruciate ligament: MNX: medial meniscectomy.

**Table 5:** Natural products of alkaloids with antiosteoarthritis function.

Compound	Source	Cell/animal model	Effect	Ref
Sinomenine	Sinomenium acutum	Chondrocytes of C57BL/6 mice induced by IL-6 and IL-1 *β*	Downregulate gene expression of iNOS, COX-2, NO, PGE2, TNF -*α*, activate the nrf2/HO-1 signaling pathways, and inhibit NF-*κ*B activity	[39]
Nicotine	Nicotiana tabacum, Solanum lycopersicum, Lycium	Chondrocytes of OA patients/*a*7-nAChR mice induced by MIA/RAW264.7 cells induced by LPS/chondrocytes of male SD rats induced by IL-1*β*	Downregulate gene expression of IL-6 and IL-1*β*, downregulate the expression of MMP-9, and increase the protein level of Bcl-2 and Bcl-xL	[41,61,92]
Ligustrazine	Rhizome of Ligusticum chuanxiong	Human OA chondrocytes	Increase SOD level, decrease ROS production and decrease MDA level, increase SOD level, decrease ROS production and decrease MDA level, increase SOX9 expression, and block NF-*κ*B pathway	[57]
Berberine	Rhizoma coptidis	Chondrocytes of male SD rats induced by IL-1*β*/male SD rats induced by ACLT and MMxs	Downregulate expression of MMP-1, MMP-3, MMP-13, and ADAMTS-5, increase S phase cells, decrease G0/G1 phase cells, upregulate *β*-catenin and cyclin D1, and downregulate GSK-3 *β*	[62,91]

ACLT: anterior cruciate ligament; MMxs, resection of medial menisci.

**Table 6:** Natural products of saponins with antiosteoarthritis function.

Compound	Source	Cell/animal model	Effect	Ref
Astragaloside	*Astragalus* membranaceus	Chondrocytes of OA patient	Inhibit IL-1b-induced production of inflammatory factors, including IL-6, TNF-a, NO, and PGE2, expression of MMP-13 and ADAMTS-5, and the activation of NF-*κ*B signaling and ameliorate the degeneration of cartilage	[42]
Hederacoside-C	The leaves of Hedera helix	Chondrocytes of male C57BL/6J mice	Reduce AGE-induced increased levels of ROS and suppress NF-*κ*B signaling pathway	[58]
Pinocembrin	Heartwood of Pinus and Cerasus pseudocerasus	Human OA chondrocytes	Downregulate the expression of MMP-1, MMP-3, and MMP-13 and inhibit TNF-a-induced phosphorylation and degradation of the NF-*κ*B inhibitor I*κ*B*α*	[68]
Icariin	Epimedium brevicornu Maxim	SW1353 chondrosarcoma cells induced by IL-1*β*/SD rats induced by ACLT	Downregulate the expression of MMP-13, reduce levels of phosphorylated p38, phosphorylated JNK, and *β*-catenin	[71]
Gentiopicroside	*Gentiana scabra* Bunge	Chondrocytes of male SD rats induced by IL-1*β*	Downregulate the expression of MMP-1, MMP-3, and MMP-13 and inhibit transduction of p38, ERK, and JNK	[72]
Ginsenoside Rg1	Panax ginseng	Human OA chondrocytes/male SD rats induced by ACLT	Inhibit IL-1*β*-induced chondrocyte gene and protein expressions of MMP-13, COX-2, and PGE2 and prevent type II collagen and aggrecan degradation, attenuated cartilage degeneration, and reduced type II collagen loss	[73]
Gypenoside	Gynostemma pentaphyllum	Human OA chondrocytes induced by IL-1*β*	Reduce MMP-3 inhibited IL-1*β*-induced NO and PGE2 production and MMP-13 expression and suppress NF-*κβ* activation	[74]
Tenuigenin	Polygala tenuifolia	Human OA chondrocytes induced by IL-1*β*	Inhibit IL-1*β*-induced NO and PGE2 production, suppress MMP-1, MMP-3, and MMP-13 expression, and inhibit IL-1*β*-induced NF-*κβ* activation, PI3K, and AKT phosphorylation	[75]
*Achyranthes bidentate* saponins	*Achyranthes bidentate*	Chondrocytes of SD rats	Suppress the activation of caspase-3, p53 protein phosphorylation and promote the expression of Bcl-xL and Bad	[93]

ACLT: anterior cruciate ligament.

**Table 7:** Natural products of others with antiosteoarthritis function.

Compound	Source	Cell/animal model	Effect	Ref
*α*-Bisabolol	Matricaria recutita, salvia and wood of candei	Human OA chondrocytes induced by AGEs/C57BL/6 mice induced by DMM	Reversed AGE-mediated overproduction of iNOS-NO, COX-2, PGE2, TNF-*α*, and IL-6	[31,48]
Tanshinone IIA, salvianolic acid B	Salvia miltiorrhiza	Male SD rats induced by ACLT/human OA chondrocytes	Downregulate iNOS, IL-1 *β*, COX-2, NO, PGE2, TNF-*α,* and ADAMTS-5	[43,44]
Astaxanthin	Haematococcus *pluvialis*, Cladophora aegagropila	Male SD rats induced by MIA/Zealand rabbits induced by ACLT	Downregulate gene expression TNF-*α*, IL-1*β*, IL-6, COX-2, and iNOS and downregulate the expression of MMP-1, MMP-3, and MMP-13	[45,77]
Delphinidin	Consolida ajacis, Solanum melongena, Commelina communis	Human chondrocytes induced by IL-1*β*/C28/I2 human chondrocytes induced by H_2_O_2_	Reduce the production of COX-2 and PGE2 and reduce production of ROS	[46,59]
Bergapten	Heracleum hemsleyanum Diels, Cnidium monnieri (L.) Cuss.	Chondrocytes of SD rats induced by IL-1*β*	Ameliorate expression of inflammatory cytokines and mediators, including Il‐1, Il‐6, TNF‐*α*, Cox‐2, and MMP‐13, maintain chondrocyte phenotype, and promote the secretion of cartilage‐specific extracellular matrix by activating the ANP32 A/ATM signaling pathway	[47]
Schisandrin	Schisandra chinensis	Chondrocytes of SD rats/male SD rats induced by ACLT	Suppress the production of NO inhibit cartilage matrix catabolic enzymes including MMPs and ADAMTS-5, increase the expression collagen II, aggrecan, and Sox9, and suppress MAPK and NF-*κ*B signal pathways and PGE2, iNOS, and COX-2	[76]
Psoralen	*Psoralea* corylifolia	Chondrocytes of SD rats induced by TNF-*α*/chondrocytes of SD rats	Decrease the gene expression of MMPs and ILs and upregulate gene expression of cyclin D1	[81,94]
Tanshinone I	Salvia miltiorrhiza	Chondrocyte line chon-001 induced by IL-1*β*/C57BL/6 mice induced by ACLT	Reverse collagen II, aggrecan degradation, SOX11 downregulation, and MMP-13 and p–NF–*κ*B upregulation	[78]
Shikonin	Lithospermum erythrorhizon	Chondrocytes of Zealand rabbits induced by IL-1*β*/Zealand rabbits induced by ACLT	Inhibit the gene expression of MMP-1, MMP-3, and MMP-13 and increase that of TIMP-1	[79]
Polyoxypregnane xglycoside	Dregea volubilis	Human OA chondrocytes and porcine induced by H_2_O_2_	Inhibit MMPs gene and protein expression, reverse effect of IL-1*β*-inhibited expression of type II collagen	[80]

ACLT: anterior cruciate ligament; DMM: destabilization of medial meniscus; MIA: injection of sodium iodoacetate.

## Data Availability

The data used to support the results of this study are included within the article. The data used to support the findings of this study are also available from the corresponding author upon request.
